# Comparison of the respiratory effects of commonly utilized general anaesthesia regimes in male Sprague-Dawley rats

**DOI:** 10.3389/fphys.2023.1249127

**Published:** 2023-09-18

**Authors:** Bence Ballók, Álmos Schranc, Ibolya Tóth, Petra Somogyi, József Tolnai, Ferenc Peták, Gergely H. Fodor

**Affiliations:** ^1^ Department of Medical Physics and Informatics, Albert Szent-Györgyi Medical School, University of Szeged, Szeged, Hungary; ^2^ Unit for Anaesthesiological Investigations, Department of Anaesthesiology, Pharmacology, Intensive Care, and Emergency Medicine, University of Geneva, Geneva, Switzerland; ^3^ Department of Cell Biology and Molecular Medicine, Albert Szent-Györgyi Medical School, University of Szeged, Szeged, Hungary

**Keywords:** respiratory mechanics, general anaesthesia, animal model, lung function, rat

## Abstract

**Background:** Respiratory parameters in experimental animals are often characterised under general anaesthesia. However, anaesthesia regimes may alter the functional and mechanical properties of the respiratory system. While most anaesthesia regimes have been shown to affect the respiratory system, the effects of general anaesthesia protocols commonly used in animal models on lung function have not been systematically compared.

**Methods:** The present study comprised 40 male Sprague-Dawley rats divided into five groups (*N* = 8 in each) according to anaesthesia regime applied: intravenous (iv) Na-pentobarbital, intraperitoneal (ip) ketamine-xylazine, iv propofol-fentanyl, inhaled sevoflurane, and ip urethane. All drugs were administered at commonly used doses. End-expiratory lung volume (EELV), airway resistance (Raw) and tissue mechanics were measured in addition to arterial blood gas parameters during mechanical ventilation while maintaining positive end-expiratory pressure (PEEP) values of 0, 3, and 6 cm H_2_O. Respiratory mechanics were also measured during iv methacholine (MCh) challenges to assess bronchial responsiveness.

**Results:** While PEEP influenced baseline respiratory mechanics, EELV and blood gas parameters (*p* < 0.001), no between-group differences were observed (*p* > 0.10). Conversely, significantly lower doses of MCh were required to achieve the same elevation in Raw under ketamine-xylazine anaesthesia compared to the other groups.

**Conclusion:** In the most frequent rodent model of respiratory disorders, no differences in baseline respiratory mechanics or function were observed between commonly used anaesthesia regimes. Bronchial hyperresponsiveness in response to ketamine-xylazine anaesthesia should be considered when designing experiments using this regime. The findings of the present study indicate commonly used anaesthetic regimes allow fair comparison of respiratory mechanics in experimental animals undergoing any of the examined anaesthesia protocols.

## Introduction

The assessment of pulmonary function and mechanics in experimental animals, particularly rodents, typically requires anaesthesia of the animal as opposed to practically all similar measurements in human subjects (Bates, 2017). General anaesthesia itself has a dose-dependent influence on the respiratory system (Fagerlund et al., 2020), predominantly due to respiratory depression or changes in breathing patterns caused by perturbed control of breathing and loss of respiratory muscle tone (Hans et al., 2009). This can lead to rapid formation of atelectasis and airway closure resulting in reduced functional residual capacity, hypoxia, hypercapnia and increased intrapulmonary shunting (Brismar et al., 1985; Hedenstierna and Edmark, 2005; Hans et al., 2009). These blood gas abnormalities may also have bronchial and pulmonary vascular effects, which ultimately alter respiratory mechanics (Hedenstierna and Edmark, 2005; Hans et al., 2009). These effects can be mitigated by mechanical ventilation, thereby preventing most of these complications (Brismar et al., 1985; Tokics et al., 1987).

Agents used for general anaesthesia can have various direct and indirect effects on the respiratory system. Several previous studies have provided evidence of the bronchodilator effects of volatile anaesthetics in animal and human models (Mitsuhata et al., 1994; Habre et al., 1997; Habre et al., 2001; Balogh et al., 2017). Other studies have described serious respiratory adverse effects of other anaesthetic drugs, such as xylazine-induced pulmonary oedema and surfactant dysfunction (Amouzadeh et al., 1991; Adam et al., 2022), although xylazine is commonly used for sedation alone or for general anaesthesia in combination with ketamine. Studies of the effects of other anaesthesia agents on respiratory function and mechanics have reported ambiguous and contradicting results.

Despite the lack of clarity, the effects of anaesthesia protocols commonly used in animal models on lung function have not been systematically compared. Accordingly, the present study aimed to assess the respiratory effects of agents frequently used for general anaesthesia in rats as anaesthesia is used in most rodent models of respiratory disorders.

## Materials and methods

### Ethical approval

The present study received ethical approval from the National Food Chain Safety and Animal Health Directorate of Csongrád County, Hungary (no. XXXII./2110/2019) on 16 December 2019. The study described in this manuscript was conducted in compliance with the guidelines of the Scientific Committee of Animal Experimentation of the Hungarian Academy of Sciences (updated Law and Regulations on Animal Protection: 40/2013 [II. 14.], the Government of Hungary) and the European Union Directive 2010/63/EU on the protection of animals used for scientific purposes. Results were reported according to the ARRIVE guidelines (Percie du Sert et al., 2020).

### Group allocation according to anaesthesia induction and maintenance

Forty male Sprague-Dawley rats (280–540 g, CD^®^ IGS Rat, Charles River, Germany; 8–14 weeks of age) were randomly allocated to one of five experimental groups (*N* = 8 in each group). Groups were assigned according to the anaesthetic agent used for induction: group PB were administered an intraperitoneal (ip) bolus of sodium pentobarbital (45 mg/kg, (Wixson and Smiler, 1997)), group KX were administered an ip injection of ketamine (60 mg/kg) and xylazine [10 mg/kg, (Wixson and Smiler, 1997)), group U were administered an ip injection of urethane (1,000 mg/kg, (Wixson and Smiler, 1997)] and groups PF and group S were administered inhalational sevoflurane. Those animals were placed in a body box filled with room air and 5% sevoflurane. Inhalational sevoflurane at a concentration of 1–2 MAC (2.2%–3.5% (Obal et al., 2001)) was then continuously administered through a face mask and then using a small animal ventilator (Model 683, Harvard Apparatus, South Natick, MA, United States) through a tracheostomy. Animals were surgically prepared, and anaesthesia was maintained according to group allocation. Anaesthesia was maintained using repeated intravenous (iv) boluses of sodium pentobarbital (12 mg/kg, every 30 min) in group PB (Wixson and Smiler, 1997), applying repeated ip boluses of ketamine (60 mg/kg) and xylazine (10 mg/kg) every 30 min in group KX (Wixson and Smiler, 1997), using a continuous iv infusion of propofol 30 mg/kg/h) and fentanyl (5 μg/kg/h) in group PF (Wixson and Smiler, 1997) or continuous inhalation of sevoflurane at a dose of 1–2 MAC (2.2%–3.5%, (Obal et al., 2001)) through a tracheostomy using a ventilator in group S. Additional maintenance of anaesthesia following induction was not required in group U (Wixson and Smiler, 1997).

### Animal preparation

Following anaesthesia induction, subcutaneous injection of lidocaine (2–4 mg/kg) was locally administered and tracheostomy was performed using a 2.5-mm metal cannula (tracheal cannula with Luer end, 2.5 mm OD, # 732725, Harvard Apparatus, South Natick, MA, United States). Mechanical ventilation was initiated with a rodent ventilator using room air at a tidal volume of 10 ml/kg using a frequency that achieved normocapnia. End-tidal CO_2_ was monitored using a sidestream rodent capnograph (Type 340, Harvard Apparatus, South Natick, MA, United States). The left femoral artery and vein were cannulated using a polyethylene tube (Abbocath 22G). The arterial line was connected to a pressure transducer (MLT0380 Reusable blood pressure Transducer, ADInstruments, Dunedin, New Zealand) and was also used to draw blood samples for arterial blood gas measurements (epoc Reader and Host, Epocal Inc., Ottawa, ON, Canada). The femoral venous line was used for delivery of the anaesthetic agent, of pipecuronium a neuromuscular blocking agent at 0.1 mg/kg every 30 min and for administration of the MCh infusion. As a continuous iv infusion was required for anaesthesia maintenance in group PF, an additional venous catheter was inserted into the right jugular vein in the animals of this group. Animals were placed in the supine position on a heating pad with a rectal thermometer connected and set to 37.0°C ± 0.5°C (Model 507223F, Harvard Apparatus, South Natick, MA, United States). Vital parameters (ECG, arterial blood pressure, body temperature, and exhaled CO_2_) were monitored and recorded using a data collection and acquisition system (Powerlab 8/35 and Labchart, ADInstruments, Dunedin, New Zealand).

### Measurement of end-expiratory lung volume (EELV)

EELV was measured using a custom-made whole-body plethysmography box as detailed previously (Janosi et al., 2006). Briefly, the animal was placed in a sealed plexiglas box and ventilated normally. During measurements, the trachea and box were closed for 10–15 s at end-expiration and pressures inside the box (Pbox) and the trachea (Ptr) were measured during spontaneous breathing efforts of the animal against the closed trachea. Measurements were performed at different levels of positive end-expiratory pressure (PEEP). EELV was calculated from simultaneously measured pressure signals by applying the Boyle–Mariotte law. To compensate for differences in body size between animals, EELV values were normalised to body mass (Janosi et al., 2006).

### Measurement of respiratory mechanics

Respiratory mechanical parameters were measured by the forced oscillatory method as described in detail previously (Petak et al., 1997; Fodor et al., 2019; Schranc et al., 2021). Briefly, a short (8 s) end-expiratory apnoea was induced, and the tracheal cannula was connected to a loudspeaker-in-box system by turning a three-way tap. The loudspeaker delivered a small amplitude (<±1 cmH_2_O) pseudorandom forcing pressure signal consisting of 23 non-integer multiples of a 0.25-Hz fundamental frequency between 0.5 and 20.75 Hz through a polyethylene wave-tube (100 cm length, 2 mm internal diameter). Lateral pressures on both ends of the wave-tube were measured (P1 and P2) using two identical differential pressure transducers (24PCEFA6D, Honeywell, Charlotte, NC, United States), low-pass filtered at 25 Hz, and digitised using an analogue-digital converter of a data acquisition board (USB-6211, National Instruments, Austin TX) at a sampling rate of 256 Hz. Pressure transfer functions (P1/P2) were calculated with fast Fourier transformation (4-s time windows and 95% overlapping) from each 6-s recording. The input impedance of the respiratory system (Zrs) was calculated as the load impedance of the wave-tube from the recordings (Franken et al., 1981), and at least three consecutive and consistent Zrs measurements were ensemble-averaged under each experimental condition. The input impedance of the endotracheal tube and the connections had previously been determined and subsequently subtracted from each Zrs spectrum.

The constant-phase model was fitted to the Zrs spectra by minimizing the relative difference between the measured and modelled impedance data (Hantos et al., 1992). The model included frequency-independent resistance (Raw) and inertance (Iaw). The model also contained a constant-phase tissue compartment consisting of tissue damping (G) and tissue elastance (H). G describes the dissipative properties (damping and resistance) while H describes the stiffness (elastance) of the respiratory tissues (lung and chest wall). Raw and Iaw describe the flow resistance and inertance of mainly the conducting airways as the contribution of the chest wall is minimal on these parameters (Petak et al., 1998).

### Study protocol

**FIGURE 1:**
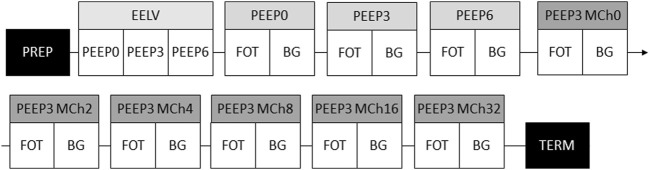
Schematic of the experimental protocol. PEEP, positive end-expiratory pressure; PREP, anaesthesia induction, animal preparation and surgery; EELV, assessment of end-expiratory lung volume; FOT, respiratory mechanical measurements; BG, arterial blood gas sampling; MCh, methacholine challenge; TERM, termination of the animal.

The experimental scheme is illustrated in Figure 1. Following animal preparation, EELV was measured using whole-body plethysmography with increasing levels of PEEP at 0, 3, and 6 cm H_2_O, respectively. Animals were then removed from the body box and neuromuscular blockade was initiated. Respiratory mechanics were determined using forced oscillations while maintaining the same PEEP levels as with EELV (0, 3, and 6 cm H_2_O, again in an increasing order). Arterial blood samples were collected and analysed at each PEEP level to assess the partial pressure of oxygen (PaO_2_), partial pressure of carbon dioxide (PaCO_2_), pH and serum glucose level. Finally, the responsiveness of the respiratory system to an exogeneous bronchoconstrictor stimulus was measured at baseline and then with administration of a continuous iv infusion of methacholine (MCh) at increasing doses (4, 8, 16, and 32 μg/kg/min) while maintaining a PEEP of 3 cm H_2_O. All measurements were performed under anaesthesia maintained in accordance with group allocation. Animals were euthanized at the end of the experiment using an iv bolus of 200 mg/kg pentobarbital sodium.

### Primary outcome variables

Primary outcome variables were EELV, respiratory mechanical parameters (Raw, G and H), and respiratory responsiveness (ED100_Raw_ defined as the MCh dose required for a 100% increase in Raw).

### Secondary outcome variables

Secondary outcomes were blood gas parameters (PaO_2_, PaCO_2_, pH and serum glucose) and baseline haemodynamic status measured with mean arterial pressure (MAP) and heart rate (HR).

### Statistical analyses

Data are presented as mean ± SD for normally distributed variables. Normality was checked using the Shapiro-Wilk test. Two-way repeated-measures analyses of variance (ANOVA) with Holm-Sidak *post hoc* tests were performed to assess the effects of various anaesthesia regimes on PEEP-dependence or MCh dose-dependence of respiratory or vital parameters. One-way ANOVA was applied to test for anaesthesia-related differences in the responsiveness of the respiratory system. Airway resistance was the primary outcome variable for estimating the sample size required for repeated-measures ANOVA with a power of 0.8 and an alpha of 0.05. Based on previous data (Sudy et al., 2019), the power analysis revealed that at least six animals were required in each protocol group to detect a 40% difference as statistically significant. Two-tailed *p*-values less than 0.05 were considered statistically significant.

## Results

**FIGURE 2:**
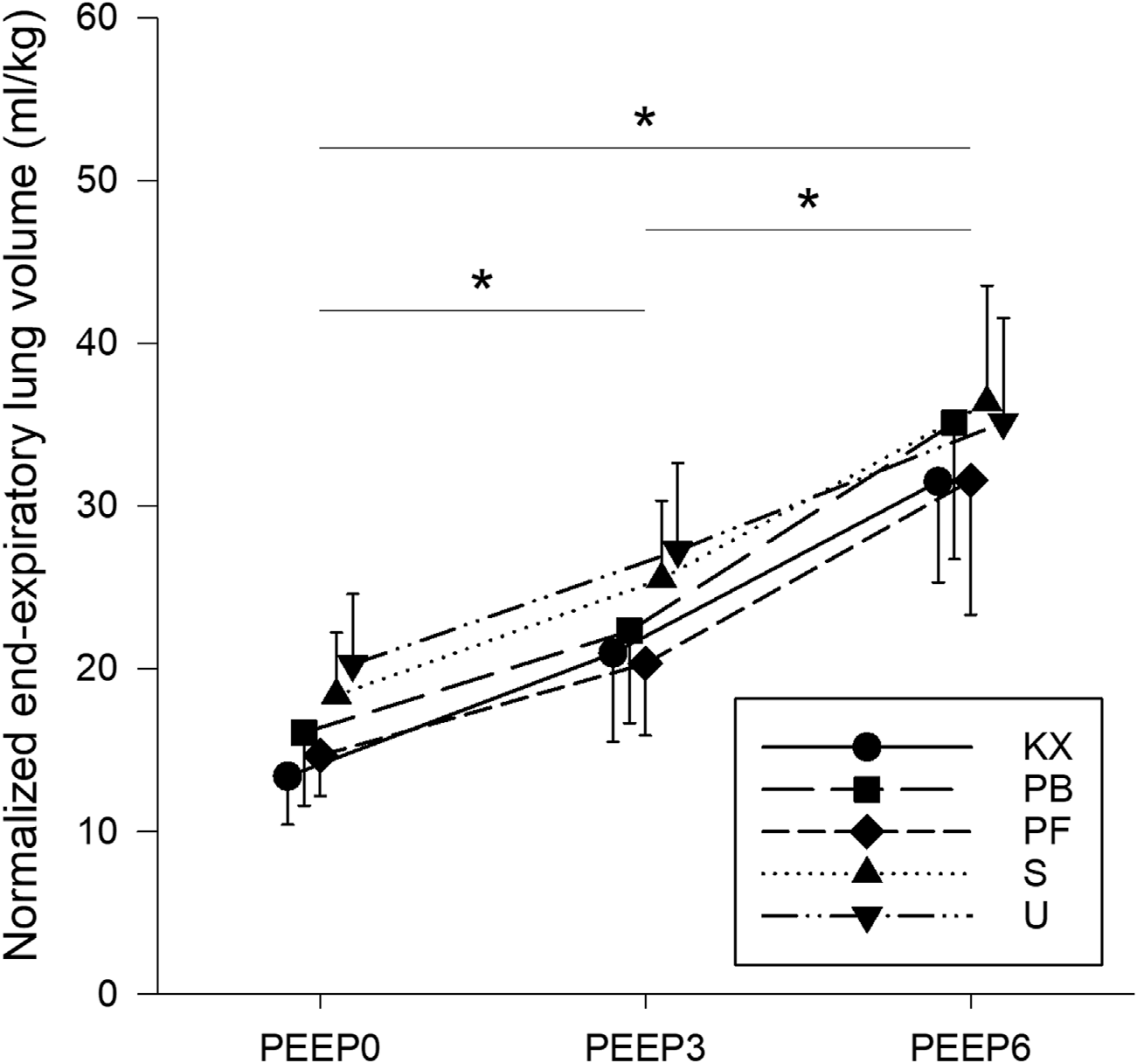
End-expiratory lung volumes normalised to body mass. KX, ketamine-xylazine; PB, pentobarbital sodium; PF, propofol-fentanyl; S, sevoflurane; U, urethane; PEEP, positive end-expiratory pressure. *p < 0.05 for all groups. *N* = 8 in each group.

The body masses of rats are demonstrated in Table 1. No significant differences in body masses were observed between groups (*p* = 0.92). EELV was normalised to body mass due to a wide distribution of body masses within groups. EELV values normalised to body mass are shown in Figure 2. Significant increases in EELV were observed at higher PEEP levels in all groups (*p* < 0.001 for all), with no differences observed between any of the experimental groups (*p* > 0.10).

**TABLE 1:** Body masses of the rats in each group. Data is displayed as mean ± SD. No significant differences were observed. *N* = 8 in each group.

Group KX	Group PB	Group PF	Group S	Group U
405.0 ± 74.4 g	395.6 ± 86.2 g	421.9 ± 56.8 g	420.0 ± 52.4 g	405.0 ± 43.3 g

**FIGURE 3:**
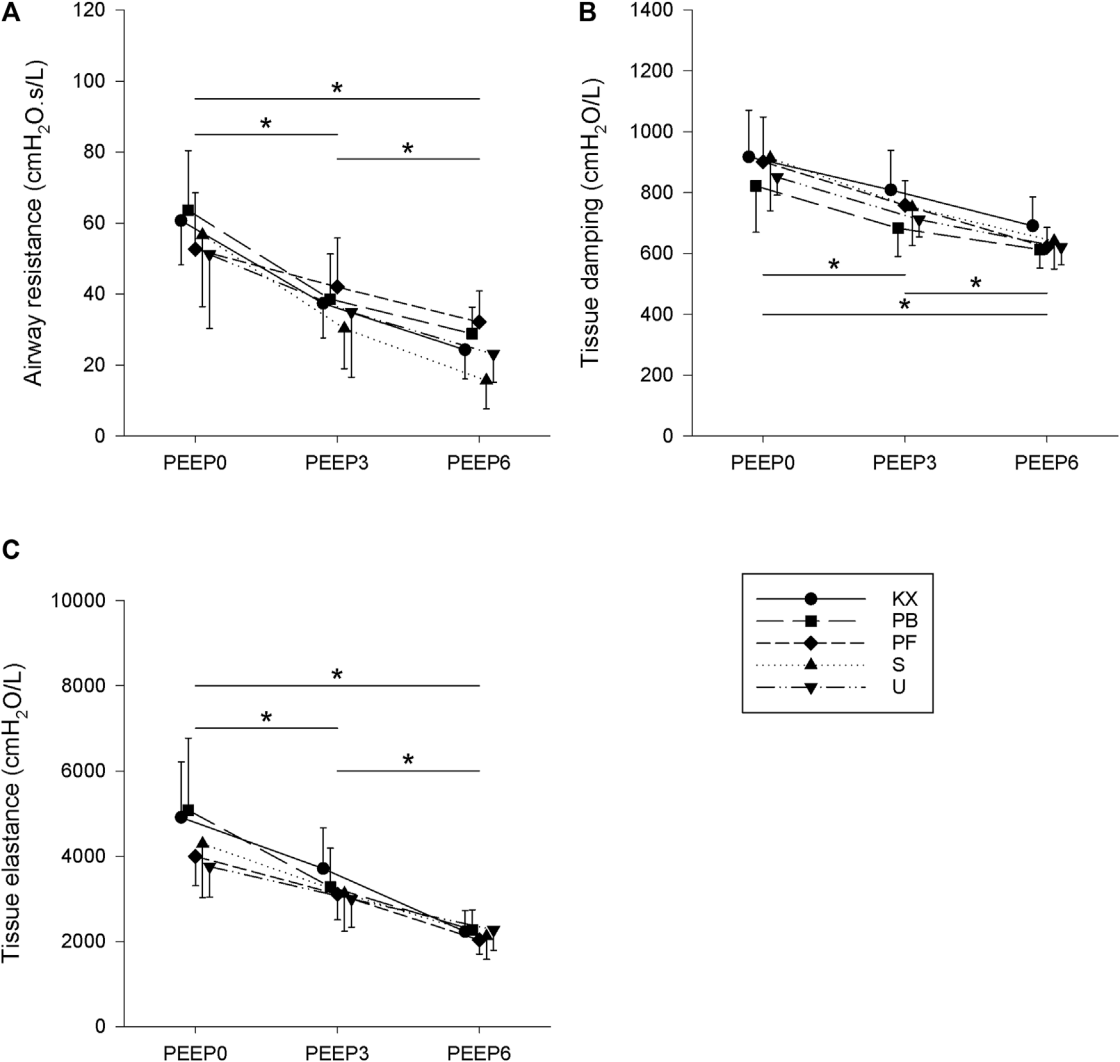
Baseline respiratory mechanical parameters. **(A)** Airway resistance. **(B)** Tissue damping. **(C)** Tissue elastance. KX, ketamine-xylazine; PB, pentobarbital sodium; PF, propofol-fentanyl; S, sevoflurane; U, urethane; PEEP, positive end-expiratory pressure. *p < 0.05 for all groups. *N* = 8 in each group.

Baseline respiratory mechanical parameters are illustrated in Figure 3. Raw significantly decreased with increasing PEEP (*p* < 0.001 for all), with no differences observed between any of the experimental groups (*p* = 0.54). Similar trends were observed in terms of the tissue mechanical parameters G and H, with marked effects of PEEP (*p* < 0.001 for all) and no differences between experimental groups (*p* = 0.36 for G and *p* = 0.33 for H).

**FIGURE 4:**
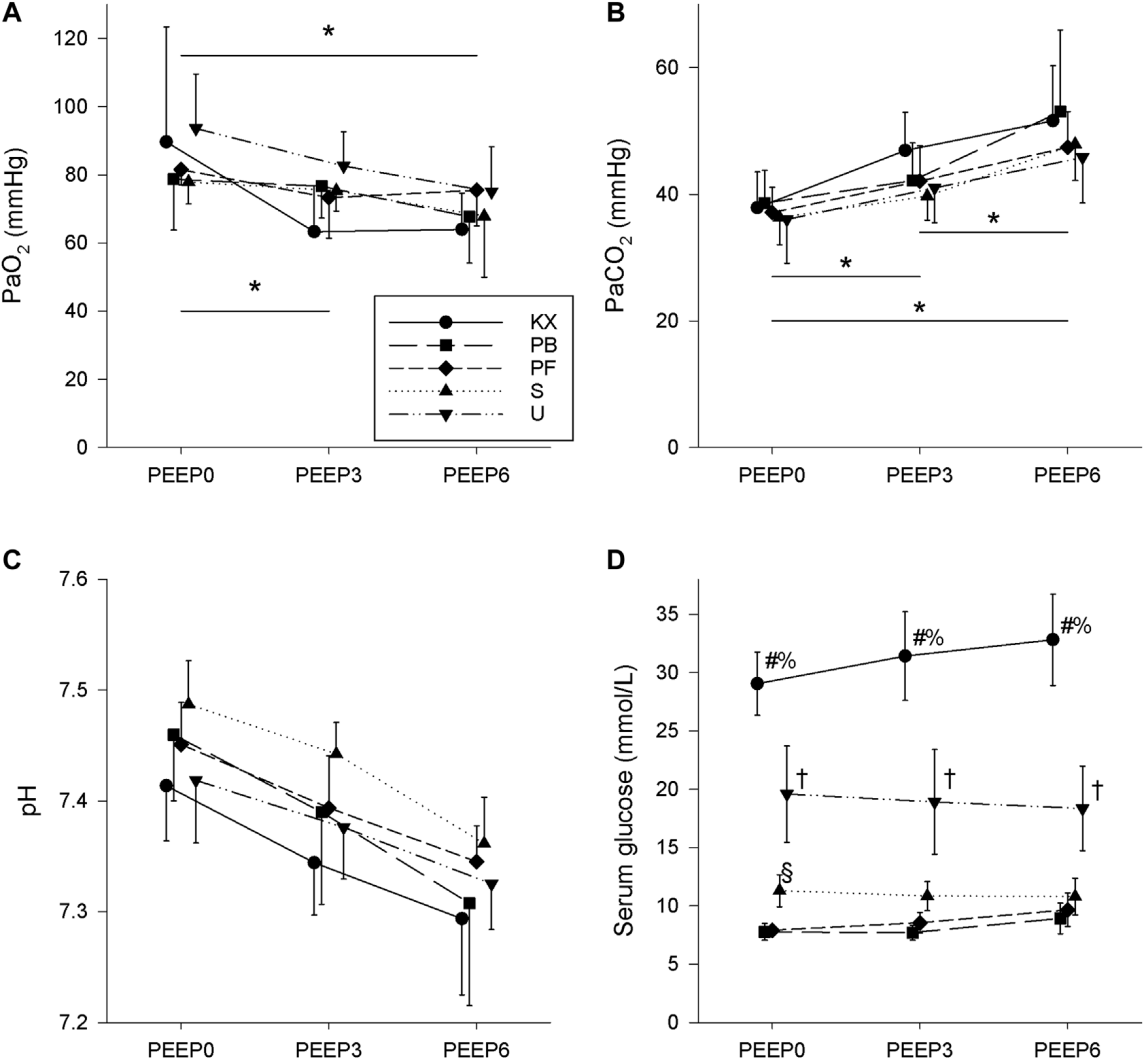
Baseline arterial blood gas parameters. **(A)** Partial pressure of O_2_ (PaO_2_). **(B)** Partial pressure of CO_2_ (PaCO_2_). **(C)** Arterial pH. **(D)** Serum glucose. KX, ketamine-xylazine; PB, pentobarbital sodium; PF, propofol-fentanyl; S, sevoflurane; U, urethane; PEEP, positive end-expiratory pressure. *p < 0.05 for all groups; #*p* < 0.05 for group KX vs. other groups; †*p* < 0.05 for group U vs. other groups; §*p* < 0.05 for group S vs other groups; % *p* < 0.05 vs. other PEEP levels in group KX. No significant differences in arterial pH values were observed. *N* = 8 in each group.

Baseline blood gas parameters are depicted in Figure 4. No significant differences in PaO_2_ values were observed between groups (*p* = 0.27); however, a negative PEEP-dependence was observed (*p* < 0.001). Increased PEEP caused significant increases in PaCO_2_ in all groups (*p* < 0.05 in all), with no differences observed between any of the experimental groups (*p* = 0.52). Increasing PEEP levels had no effect on arterial pH (*p* = 0.63), with no differences in arterial pH values observed between experimental groups (*p* = 0.84). Non-fasting serum glucose levels were not affected by PEEP and were within the normal range in groups PB and PF (*p* > 0.05 in all groups). While PEEP had no significant effect on the serum glucose levels in group S (*p* > 0.50), significantly higher serum glucose levels were observed in group S at a PEEP of 0 cm H_2_O compared to groups PB and PF (*p* = 0.03 and *p* = 0.02, respectively). There was a tendency for this difference to be also present at a PEEP of 3 cm H_2_O (*p* = 0.06 for PB and *p* = 0.15 for PF), while serum glucose levels in group S did not differ from those in groups PB or PF at a PEEP of 6 cmH_2_O (*p* = 0.41 and *p* = 0.61, respectively). Of note, serum glucose levels remained within the normal range for most animals in group S. Conversely, markedly elevated serum glucose levels that exceeded the normal range were observed in groups KX and U, and these values were significantly higher than in all other groups (*p* < 0.001). While PEEP had no effect on the above-normal glucose levels in group U (*p* > 0.05), a significant increase in serum glucose levels with increasing PEEP was observed in group KX (*p* < 0.02).

**FIGURE 5:**
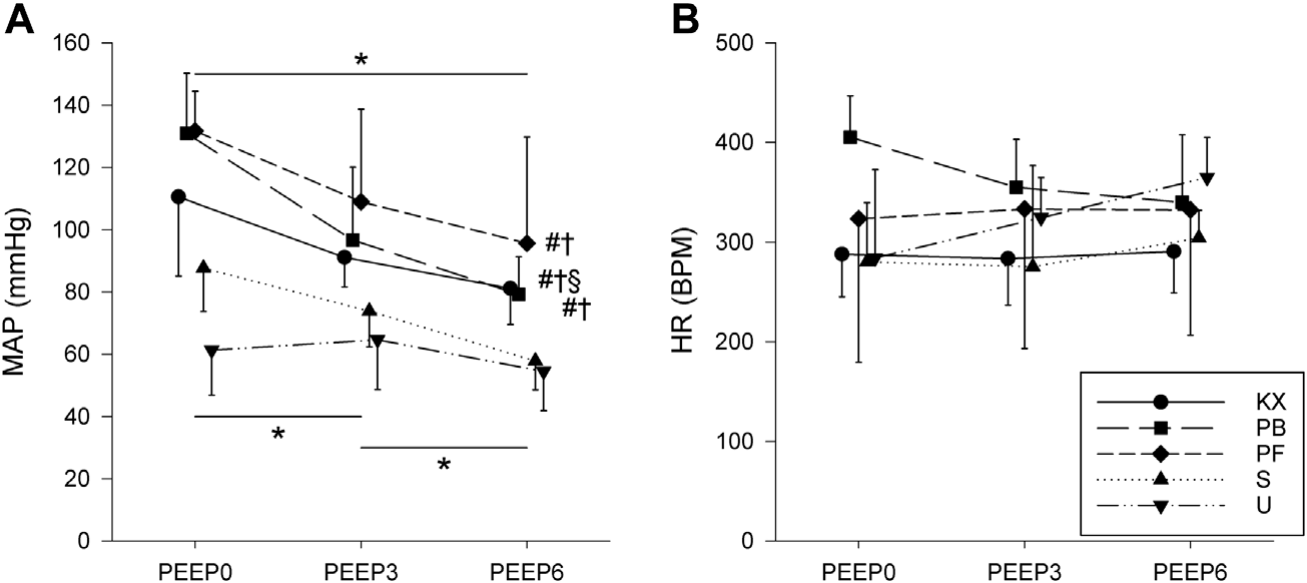
Baseline haemodynamic parameters. **(A)** Mean arterial blood pressure (MAP). **(B)** Heart rate (HR). KX, ketamine-xylazine; PB, pentobarbital sodium; PF, propofol-fentanyl; S, sevoflurane; U, urethane; PEEP, positive end-expiratory pressure. *p < 0.05 for all groups; #*p* < 0.05 versus group S; †*p* < 0.05 versus group U; §*p* < 0.05 versus group PF. No significant differences in HR were observed. *N* = 8 in each group.

Baseline haemodynamic parameters (MAP and HR) are shown in Figure 5. HR was stable in all groups (*p* = 0.57) regardless of the PEEP applied, with no significant differences between experimental groups (*p* = 0.18). MAP decreased in response to elevated PEEP levels in all groups (*p* < 0.01). Animals in groups S and U had significantly lower MAP values compared to the other three groups (*p* < 0.01). Due to technical difficulties, sufficient haemodynamic data could not be obtained during the MCh provocation.

**FIGURE 6:**
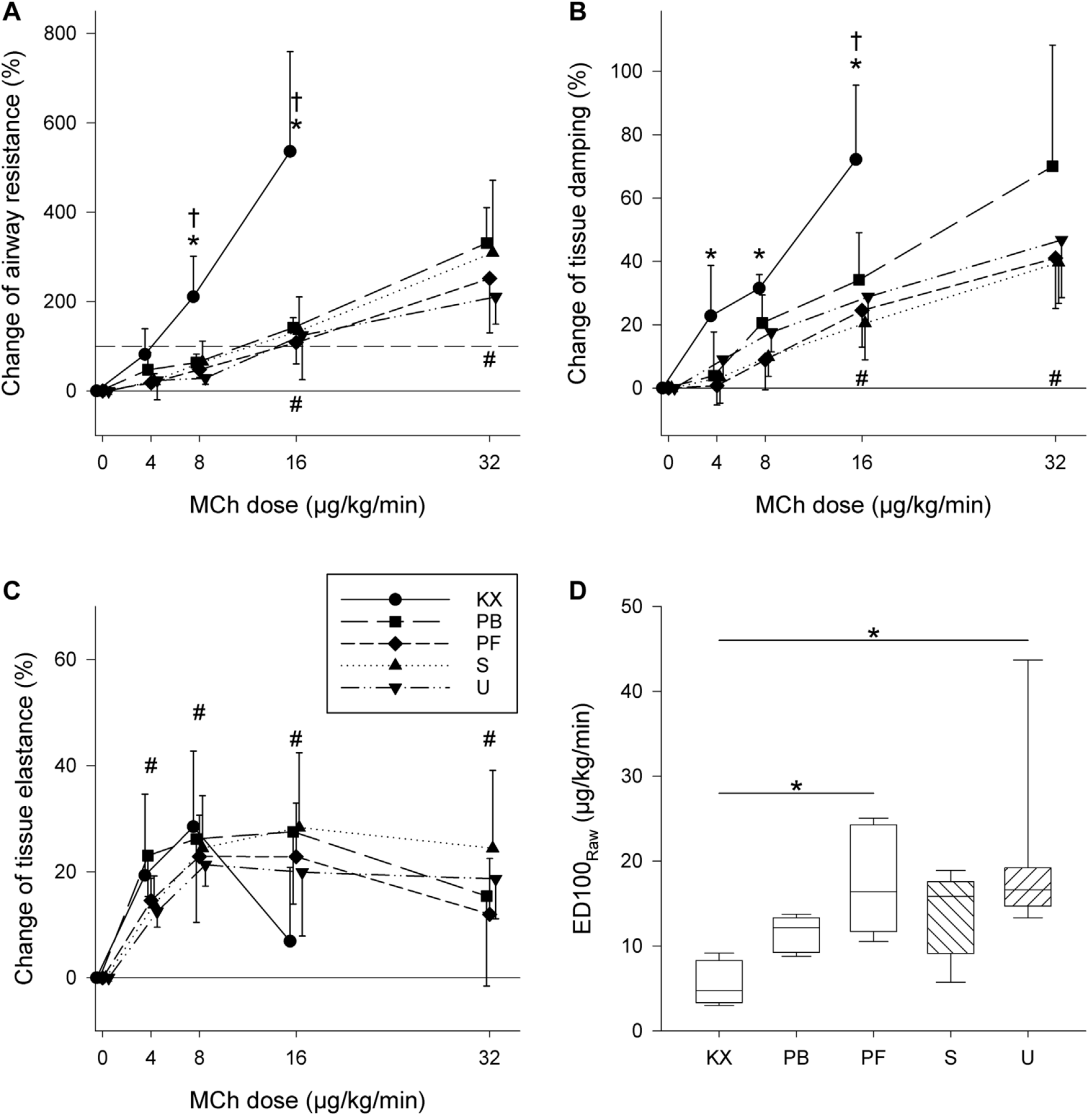
**(A–C)** Changes in respiratory mechanical parameters during intravenous methacholine (MCh) challenge **(A)** Airway resistance. **(B)** Tissue damping. **(C)** Tissue elastance. Solid grey lines indicate baseline values (0 μg/kg/min). Dashed grey lines indicate dose required for a 100% increase in airway resistance. *p < 0.05 vs baseline for group KX; †*p* < 0.05 Group KX vs. other groups; #*p* < 0.05 vs. baseline for all groups **(D)** MCh dose needed to reach 100% elevation of airway resistance (ED100_Raw_). *: *p* < 0.05. *N* = 8 in each group.

Respiratory mechanics during intravenous MCh provocation are shown in Figure 6. In terms of Raw, group KX exhibited increased reactivity compared to the other groups, with statistically significant increases observed at 8 and 16 μg/kg/min compared to baseline. Accordingly, the highest increases in Raw were in group KX compared to other groups (*p* < 0.001). Due to severe bronchoconstriction (535% ± 223% increase compared to baseline), MCh provocation was aborted in all animals in group KX at 16 μg/kg/min instead of 32 μg/kg/min in other groups. In all other groups, significant elevations in Raw were observed during MCh infusion rates of 16 and 32 μg/kg/min compared to baseline. This difference in airway reactivity was also reflected in the average values of ED100_Raw_, with the lowest ED100_Raw_ values observed in group KX (*p* < 0.03).

Significant elevations of G were also observed in group KX at MCh infusions at 4 μg/kg/min and above compared to baseline (*p* < 0.001). No differences were observed in G between the other experimental groups (*p* > 0.29), with significantly higher G values observed at MCh infusions of 16 and 32 μg/kg/min (*p* < 0.001). In terms of H, no significant differences were observed between experimental groups (*p* = 0.80), with significant elevations observed starting at MCh infusion rates 4 μg/kg/min and above (*p* < 0.001).

**FIGURE 7:**
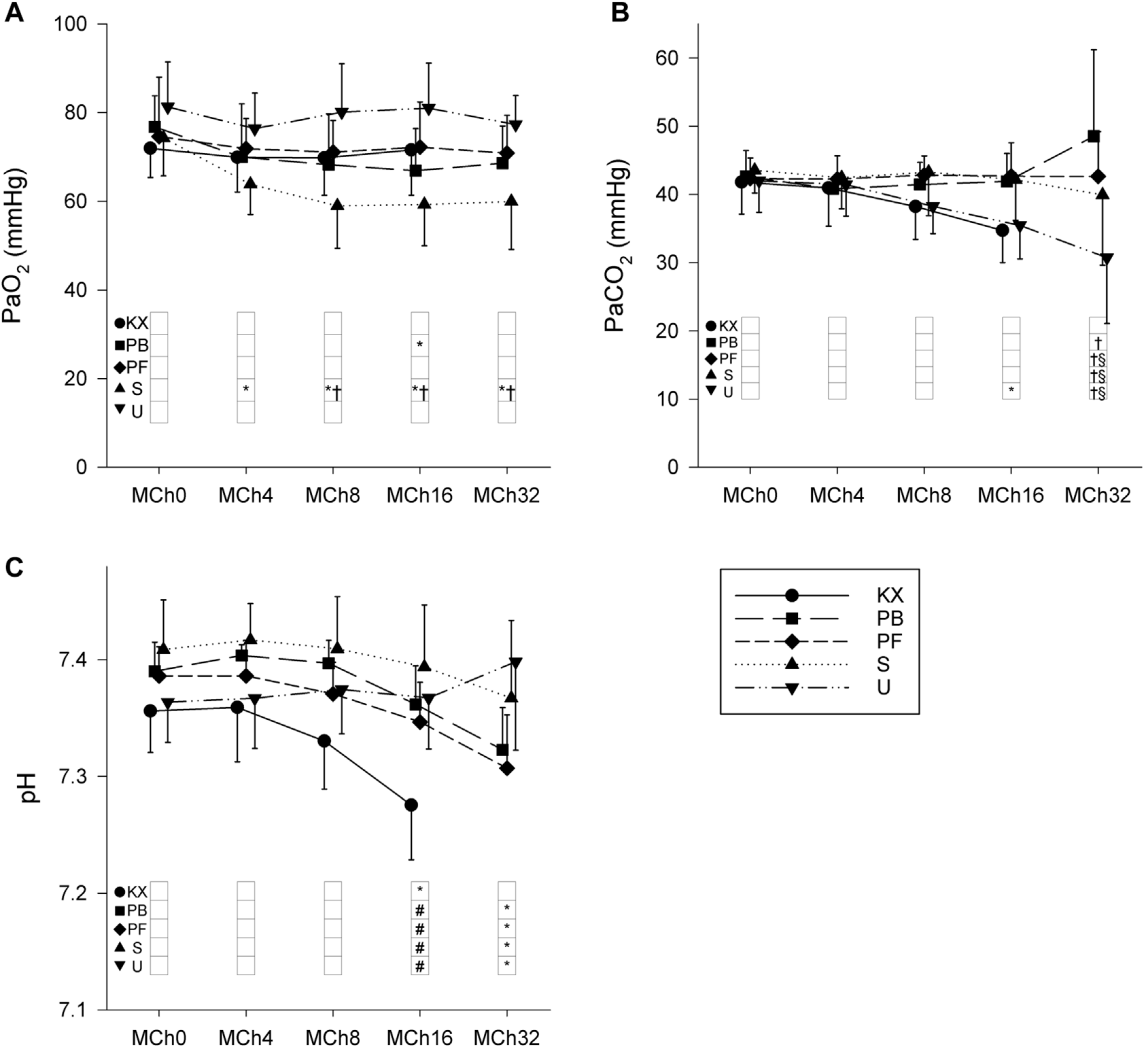
Changes in arterial blood gas parameters during intravenous methacholine (MCh) challenge. **(A)** Partial pressure of O_2_ (PaO_2_). **(B)** Partial pressure of CO_2_ (PaCO_2_). **(C)** Arterial pH. KX, ketamine-xylazine; PB, pentobarbital sodium; PF, propofol-fentanyl; S, sevoflurane; U, urethane. *p < 0.05 vs. MCh0; #*p* < 0.05 vs. Group KX; †*p* < 0.05 vs. Group U; §*p* < 0.05 vs. Group PB. *N* = 8 in each group.

Changes in blood gas parameters during iv MCh provocation are shown in Figure 7. MCh administration had no effect on PaO_2_ in most groups; however, lower PaO_2_ values were observed in group S (*p* < 0.01). Similar trends were observed with PaCO_2_ values, with decreased PaCO_2_ values observed in group U at the two highest doses of MCh (*p* < 0.04). Significant differences in arterial pH were observed at the highest dose of MCh compared to baseline in all groups (*p* < 0.04) with the exception of group U (*p* = 0.7). Despite the differences in these trends, no between-group differences were observed at any of the MCh infusion rates used.

## Discussion

The present study aimed to characterise the respiratory effects of iv sodium pentobarbital, iv propofol-fentanyl, ip ketamine-xylazine, inhaled sevoflurane and ip urethane, which are all commonly used anaesthesia regimes in rats. No significant differences in lung volume, baseline respiratory mechanics or blood gas parameters were observed between the anesthaesia regimes at three different levels of PEEP. On the contrary, significant bronchial hyperreactivity (BHR) was observed with the administration of increasing doses of iv methacholine in rats undergoing ketamine-xylazine anaesthesia. Respiratory parameters in all groups were in good agreement with data acquired previously by us in healthy animals in the same mass range and rats undergoing pentobarbital anaesthesia (Fodor et al., 2016; Sudy et al., 2019).

The lack of differences in baseline respiratory mechanical parameters between experimental groups in the present study may be attributable to a number of reasons. General anaesthesia induces respiratory depression with practically all anaesthetic agents to various extents (Brismar et al., 1985; Hedenstierna and Edmark, 2005; Hans et al., 2009), and such effects are particularly evident with the use of pentobarbital (Brown et al., 1973; Gasser et al., 1975) and propofol (Jiang et al., 2021). However, all animals were mechanically ventilated in the present study, which prevented lung volume loss and subsequently contributed to the similar EELV values observed between experimental groups. This feature of the present study in addition to physiologically low basal airway smooth muscle tone can explain the lack of the previously described bronchodilator effects of sevoflurane (Balogh et al., 2017) and propofol (Peratoner et al., 2004). While the bronchodilator effect of sevoflurane has been consistently reported in subjects with increased bronchial tone (e.g., asthma (von Ungern-Sternberg et al., 2008), chronic obstructive pulmonary disease (Volta et al., 2005) and following cardiopulmonary bypass (Balogh et al., 2017)), the bronchodilator effect of sevoflurane in normal subjects remains controversial (Mitsuhata et al., 1994; Habre et al., 1997; Correa et al., 2001). Similar to our findings, no bronchoactive properties of propofol (Brown and Wagner, 1999) or sevoflurane (Habre et al., 2001; Lele et al., 2013) were demonstrated under baseline conditions in a previous study, with propofol found to have no bronchoprotective effect against bronchoconstrictive agonists (Biddle et al., 1995; Brown and Wagner, 1999).

In contrast to previous findings under baseline conditions, anaesthesia regimes can significantly affect respiratory function during MCh-induced bronchoconstriction. Significant increases in Raw during ketamine-xylazine anaesthesia demonstrated severe BHR compared to all other anaesthetic regimes. The mechanism underlying this hyperreactivity remains unclear. Ketamine acts against cholinergic bronchoconstriction in isolated human (Gateau et al., 1989) and guinea pig bronchi (Leblanc et al., 1987; Rock et al., 1989), and sensitised rats (Zhu et al., 2007) and sheep (Brown and Wagner, 1999) *in vivo*. Previous studies on the bronchial effects of xylazine are limited; however, almost all describe a bronchodilatative effect of xylazine through activation of bronchial alpha-2 receptors (Watney et al., 1988). The discrepancy between the previously described bronchial effects of these two anaesthetics when used in isolation and our results with the combined administration of ketamine and xylazine indicates that the combination of the two drugs may result in increased airway reactivity. This increased airway resistance was also coupled with increased tissue damping and decreased arterial pH. While Raw is considered a measure of flow resistance in central conducting airways (Petak et al., 1998), G has more contributing factors. Elevation of G can most prominently indicate ventilation heterogeneity due to inhomogeneous small airway constriction and/or closure (Hantos et al., 1992; Lutchen et al., 1996). As ketamine reportedly increases the collapsibility of the lung periphery, subsequent increases in ventilation heterogeneity may explain substantial increases in G (Alves-Neto et al., 2001). While the protective effects of volatile anaesthetics (including sevoflurane) against cholinergic bronchoconstriction are well-established (Mitsuhata et al., 1994; Habre et al., 1997; Habre et al., 2001; Balogh et al., 2017), we were unable to demonstrate a protective effect of sevoflurane on bronchoconstriction in the present study. This finding may be attributable to the sustained bronchoconstriction and prolonged administration of sevoflurane in our study protocol. Indeed, sevoflurane has previously been shown to reverse cholinergic bronchoconstriction only transiently with a short duration of effect (approximately 5 min) (Schutz et al., 2004).

Apart from the respiratory effects of the applied anaesthetics, their applicability and other vital parameters also warrant discussion. Non-fasting serum glucose values were within the normal range in animals under pentobarbital, propofol-fentanyl or sevoflurane anaesthesia. However, blood sugar values above the normal range were observed under urethane and ketamine-xylazine anaesthesia. Urethane-induced hyperglycaemia may be mediated by increased sympathetic activation via alpha-2 receptors (Sanchez-Pozo et al., 1988; Aisaka et al., 1989; Takeuchi et al., 1994). The progressive hyperglycaemia observed in group KX has previously been observed in studies using ketamine (Reyes Toso et al., 1995; Zuurbier et al., 2014) or xylazine (Steffey et al., 2000) alone and in combination (Safaei et al., 2021). This finding may also be attributable to an alpha-2 receptor-mediated response, resulting in inhibition of insulin secretion (Reyes Toso et al., 1995; Safaei et al., 2021).

Normal MAP values and heart rates were observed in all groups, with the lowest mean arterial pressure values (approximately 60 mmHg) observed in animals under sevoflurane and urethane anaesthesia. This finding is in concordance with the results of previous studies describing the haemodynamic effects of these anaesthetic agents (De Wildt et al., 1983; Himori and Ishimori, 1988; Conzen et al., 1992; Brammer et al., 1993; Albrecht et al., 2014; Zuurbier, 2019; Fortea et al., 2020).

Our findings on the effects of anaesthetic agents on pulmonary system can also be considered in the context of their generalized potential to influence the function of various organ systems. Tendency for neuroprotection was proposed for volatile agents, such as sevoflurane against neuronal injury in rats (Wang et al., 2007; Schifilliti et al., 2010), barbiturates like pentobarbital (Hoffman et al., 1998; Kobayashi et al., 2007), propofol (Kobayashi et al., 2007) and ketamine (Sakai et al., 2000). Conversely, the neurological effects of urethane are different compared to other anaesthetics without a major effect on the peripheral nervous system and various subcortical areas (Maggi and Meli, 1986), thereby allowing the induction of various peripheral reflexes or characterizing cerebral processes like natural sleep (Pagliardini et al., 2013). This characteristic of urethane also results in a lesser amount of respiratory depression in spontaneously breathing animals without inducing apnoeic periods observed with other anaesthetics (Pagliardini et al., 2013). Related to the effects of short-term general anaesthesia on the liver, various studies have described either dose-dependent or idiosyncratic hepatotoxicity after using sevoflurane (Arslan et al., 2010) or propofol (Huang et al., 2014), but not with most barbiturates used for sedation or anaesthesia, or ketamine (Authors Anonymous, 2012). These hepatotoxic effects need however, a longer timeframe to develop. It is important to note the carcinogenic effects described with urethane, resulting in the development of both benignant and malignant tumours in various organs of the animals, especially after repeated injections (Du et al., 2013; Sozio et al., 2021). Thus, it is not recommended to use urethane anaesthesia in surviving animals (Wixson and Smiler, 1997).

Regarding the depth of anaesthesia, no signs of inadequate anaesthesia or inconsistency in the plane of anaesthesia were observed in any of the experimental groups, as confirmed by the stability of heart rate and blood pressure in similar experimental conditions and also before and after the administration of boluses where applicable. The depth of anaesthesia was also comparable between the different study groups, as the vital parameters of the animals were comparable with differences explainable by the known effects of the various regimes. Of note, the onset of anaesthesia was extremely long in group U (approximately 1 h), and while the dose was determined based on previous studies and published best practices, half of the animals (*N* = 4) required an additional injection of approximately 50% of the dose administered before baseline measurements.

### Limitations

The present study has several limitations that require discussion. First, the present study comprised only male Sprague-Dawley rats. While males and female mice have previously been reported to have similar baseline respiratory mechanics when adjusted for body size (Card et al., 2006), metabolic differences may influence the effects of various anaesthetics. Sex differences have been found in the metabolic rate of pentobarbital (Zambricki and Dalecy, 2004), propofol (Kodaka et al., 2005; Mukai et al., 2015), and ketamine (Saland and Kabbaj, 2018), with faster metabolism and higher doses needed in male rats or humans. While sex differences in the rate of metabolism of ketamine were also demonstrated, the effect of the actual plasma levels of various sex hormones was negligible (Saland and Kabbaj, 2018). No sex differences were found when using sevoflurane anaesthesia in humans (Kodaka et al., 2005). It is to be noted, though, that some studies have also described a faster metabolism in female humans when using propofol (Pleym et al., 2003; Maeda et al., 2016). Also, sex differences were found in mice in terms of airway responsiveness, with males showing significantly greater airway responses to methacholine provocation (Card et al., 2006).

Regarding different rat strains, differences can be expected in respiratory parameters (absolute values and responsiveness of the animals) (Boyd et al., 1982; Nakano et al., 2014; Baumann et al., 2021) and in metabolic and functional aspects of anaesthesia (Gong et al., 1998; Avsaroglu et al., 2007). However, the baseline respiratory mechanical data presented in the current study are in good agreement with those of previous studies using Wistar rats of the same size (Sudy et al., 2020; Schranc et al., 2021).

All animals used in the present study were considered to be young adults (body mass ranging between 280 g and 540 g, approximately 8–14 weeks of age). While most experiments include animals of a similar age and weight, the use of animals of differing ages may give different results due to age-related differences in anaesthesia. This can manifest as dose differences and potentially differences in respiratory function. However, the observed differences in respiratory function between the different anaesthetic regimes in the present study are likely to be genuine as age-related differences in respiratory function are predominantly related to differences in body size.

The present study comprised normal healthy rats without pathologies related to the respiratory or other organ systems. The presence of pathologic changes may alter the effects of anaesthetic agents observed in the present study, particularly when using rats as a model for respiratory diseases such as asthma, COPD or lung cancer.

The duration of the study protocol was limited. While the length of the experiments (approximately 2–2.5 h) was similar to the most frequently used experimental protocols, the examined outcomes may have differed with the use of a more prolonged experimental protocol, partly due to haemodynamical changes and heart-lung interactions.

Regarding the choice of anaesthetics, the agents and their respective doses evaluated in the present study were selected based on an extensive literature search and overview of the most commonly applied regimes. Accordingly, some anaesthetic agents were used alone (urethane, pentobarbital and sevoflurane) as they have previously been shown to have appropriate analgesic effects in rodents (De Wildt et al., 1983; Inada et al., 2020; Reimer et al., 2020). Other agents were used in combinations. Propofol alone has an insufficient analgesic effect (Goto et al., 1994) and the combination of propofol with the opioid analgesic fentanyl is routinely used in human anaesthesia. Ketamine is commonly used with xylazine in animal anaesthesia due to their synergistic analgesic and anaesthetic effects (Richardson and Flecknell, 2005). Since the effects of general anaesthesia are dose-dependent (Fagerlund et al., 2020), usage of different doses of the investigated agents can be a confounding variable and may be a subject of forthcoming investigations.

The current study aimed at characterizing the respiratory changes of animals undergoing general anaesthesia with ventilatory support. While it is known that general anaesthesia causes respiratory depression in a mostly dose-dependent manner (Fagerlund et al., 2020), changes the breathing patterns (Brismar et al., 1985; Hedenstierna and Edmark, 2005), and may result in lung volume loss via airway closure and atelectasis formation, the exact characterization of these phenomena in spontaneously breathing animals with the individual anaesthesia regimes is beyond the scope of this study.

## Summary and conclusion

The present study compared the respiratory effects of commonly used general anaesthesia regimes in ventilated male Sprague-Dawley rats. While no differences in baseline respiratory mechanics and function were observed between the different regimes, BHR developed under ketamine-xylazine anaesthesia. Accordingly, ketamine-xylazine anaesthesia may not be suitable for use in studies where bronchial reactivity is a key aspect of the protocol (e.g., sensitisation studies). Our results indicate that the baseline respiratory mechanics of ventilated rats are not influenced by commonly used anaesthesia regimes in generally administered doses, thereby allowing for a fair comparison of experimental animals undergoing different anaesthesia protocols.

## Data Availability

The raw data supporting the conclusion of this article will be made available by the authors, without undue reservation.

## Abbreviations

ANOVA, analyses of variance; BHR, bronchial hyperreactivity; ED100_Raw_, methacholine dose needed for 100% elevation of airway resistance; EELV, end-expiratory lung volume; FOT, forced oscillation technique; G, tissue damping; H, tissue elastance; HR, heart rate; Iaw, airway inertance; ip, intraperitoneal; iv, intravenous; KX, ketamine-xylazine; MAC, minimum alveolar concentration; MAP, mean arterial pressure; MCh, methacholine; PaCO_2_, partial pressure of arterial carbon dioxide; PaO_2_, partial pressure of arterial oxygen; PB, sodium pentobarbital; Pbox, pressures inside the sealed plexiglas box; PEEP, positive end-expiratory pressure; PF, propofol and fentanyl; Ptr, pressure measured inside the trachea; Raw, airway resistance; S, sevoflurane; TERM, termination of the animal; U, urethane; Zrs, input impedance of the respiratory system.
